# P16 From stewardship to strain: increasing dependence on reserve antibiotics in an intensive care setting

**DOI:** 10.1093/jacamr/dlag102.022

**Published:** 2026-06-26

**Authors:** Nesrine Hagui, Mariem Gargouri

**Affiliations:** Center of Trauma and Major Burns, Fattouma Bourguiba Hospital, Monastir, Tunisia; Center of Trauma and Major Burns, Fattouma Bourguiba Hospital, Monastir, Tunisia

## Abstract

**Background:**

Antimicrobial resistance is a major global public health threat, particularly in intensive care units where antibiotic selection pressure is high. Monitoring antibiotic consumption is a key component of antimicrobial resistance control strategies. This study aimed to analyse the quantitative and qualitative trends of systemic antibiotic use in the Anesthesia-Intensive Care Unit of the Trauma and Burn Center over a 7 year period (2018–24), using WHO standardized indicators and the AWaRe classification.

**Methods:**

We conducted a retrospective, monocentric, longitudinal pharmaco-epidemiological study over seven years. Antibiotic consumption data were extracted from the hospital pharmacy management software and expressed as Defined Daily Doses per 1000 patient-days (DDD/1000 PD). All systemic antibiotics (ATC J01) were included and classified according to the WHO AWaRe framework (Access, Watch, Reserve). Statistical analysis was descriptive, focusing on temporal trends in incidence density and proportional distribution across AWaRe groups.

**Results:**

A total of 22 779.45 DDDs were analysed over 15 181 patient-days. Overall incidence density followed a non-linear ‘U-shaped’ trend: high in 2018 (≈1957 DDD/1000 PD), decreasing to a minimum in 2021 (≈1317), then rising steadily to reach a peak in 2024 (≈2088), reflecting a marked increase in antibiotic pressure. Qualitative analysis revealed a major prescribing shift. The proportion of Access antibiotics declined from 37% in 2018 to 19% in 2024, far below WHO targets. Watch antibiotics remained predominant throughout most years (39–49%). Meanwhile, Reserve antibiotics increased sharply from 15% to 42%, becoming the leading group by the end of the study period. This rise was driven by increased use of last-resort agents: colistin (+188%), tigecycline (+781%), linezolid (+826%), and fosfomycin (+1863%). The recent introduction of ceftazidime-avibactam further illustrates growing reliance on highly specialized therapies targeting MDR organisms.

**Conclusions:**

These findings indicate critically high antibiotic selection pressure and suggest increasing endemicity of MDR bacteria. Urgent implementation of a structured antimicrobial stewardship programme—integrating prescription audits, therapeutic de-escalation, rapid diagnostics, and strengthened infection control measures—is essential to preserve future anti-infective efficacy.

